# A confounding coincidence: epidural anesthesia and paraplegia due to intramedullary tuberculoma in a patient who underwent cholecystectomy

**DOI:** 10.1186/1471-2253-14-100

**Published:** 2014-11-05

**Authors:** Changyi Wu, Ying Zhang, Jianmin Xu

**Affiliations:** Department of Anesthesiology, Peking University Third Hospital, Peking University, Beijing, 100191 China; Department of Rehabilitation, China Rehabilitation Research Center, Boai Hospital, School of Rehabilitation Medicine, Capital Medical University, Beijing, 100068 China; Department of Radiology, China Rehabilitation Research Center, Boai Hospital, Beijing, 100068 China

**Keywords:** Epidural anesthesia, Intramedullary spinal tuberculoma, Paraplegia

## Abstract

**Background:**

Paraplegia associated with epidural anesthesia or caused by intramedullary spinal tuberculoma is rare but catastrophic. We present a case of paraplegia following epidural anesthesia in a patient with an undiagnosed intramedullary spinal tuberculoma.

**Case presentation:**

A 42-year-old man developed paraplegia after an open cholecystectomy under epidural anesthesia. Spinal cord infarction, acute transverse myelitis, and intramedullary neoplasms were ruled out by histopathologic examination, and intramedullary spinal tuberculoma at the T_6_–T_7_ level was identified. Despite surgical treatment and subsequent antituberculous therapy, the patient retained some disability attributable to the delay in diagnosis.

**Conclusion:**

Physicians should be aware of coexisting disease as a cause of paraplegia following procedures using epidural anesthesia. Magnetic resonance imaging is the most sensitive diagnostic test, although it is still difficult to differentiate spinal cord infarction, myelitis, intramedullary spinal tuberculoma, and neoplasms from imaging features alone.

## Background

Epidural anesthesia is widely used during surgical procedures. A rare but devastating complication after epidural anesthesia, paraplegia may result from extradural hematoma or abscess, arterial and venous infarction of the spinal cord, chemical toxicity, or pre-existing diseases [1]. We present a case of paraplegia following epidural anesthesia in a patient with an undiagnosed intramedullary spinal tuberculoma.

## Case presentation

A 42-year-old man with cholecystolithiasis had an open cholecystectomy under epidural anesthesia at a local hospital 2 months prior to admission. He weighed 72 kg and was 173 cm tall, and had no significant medical history. During the operation his vital signs were stable and no hypotension was observed. The epidural catheter was inserted via the T_7_–T_8_ interspace, and passed upward to the upper thoracic levels. During insertion of the catheter he felt a shooting pain over the right lower leg, which disappeared immediately. Five milliliters of 2% lidocaine was injected as a test dose. Five minutes after the test-dose injection, 15 mL of 2% lidocaine (with adrenaline 1:200,000) was given incrementally, obtaining a T_4_ sensory level. The operation was uneventful. At the end of surgery, the epidural catheter was removed.

On the fifth postoperative day, the patient complained of numbness and a paresis of the right leg, which spread to the left leg. Thereafter he was evaluated by an anesthetist and a neurologist. Neurologic examinations showed that sensory deficit was below the T_7_ level, muscle power was grade 2/5 over the right lower limb and 4/5 over the left lower limb, deep tendon reflexes of both lower limbs were increased, and the Babinski sign was bilaterally positive. He had bladder incontinence and constipation, and his positional sense of the left leg was impaired. Emergency thoracic computed tomography (CT) ruled out disk herniation, epidural abscess, and epidural hematoma. Both thoracic magnetic resonance imaging (MRI) studies and motor-evoked potentials recorded from the lower limbs were normal. Therefore, transverse myelitis was diagnosed and the patient was administered dexamethasone and γ-globulin. Two months after initiation of therapy, the patient had not yet shown any neurologic improvement, and was transferred to our hospital for further treatment.

**Figure 1:**
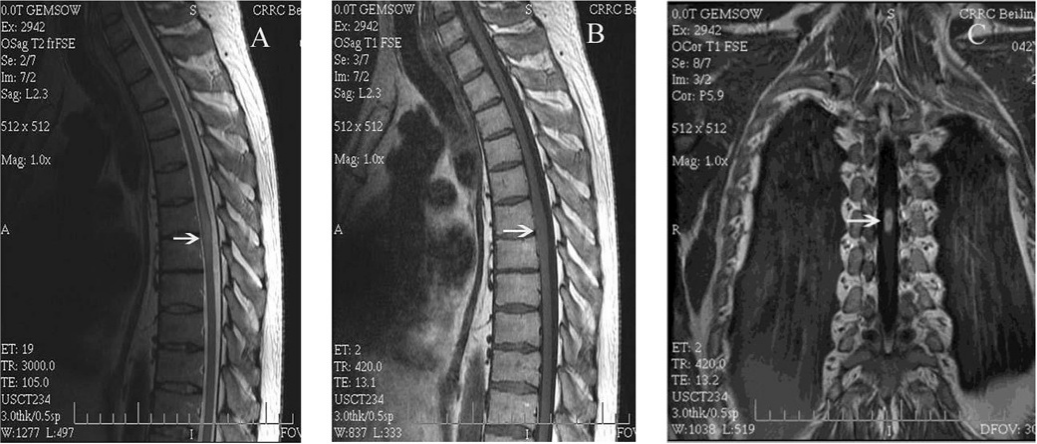
**The second MRI images of the thoracic spine. A)** Sagittal T_2_-weighted MRI image shows a hyperintense lesion expanding the cord at the T_6–7_ spinal level. **B)** Sagittal T_1_-weighted MRI image shows an isointense lesion expanding the cord at the T_6–7_ spinal level. **C)** Coronal T_1_-weighted MRI image shows a ring-like enhancing intramedullary lesion (16 × 7 × 6 mm) with gadolinium.

**Figure 2:**
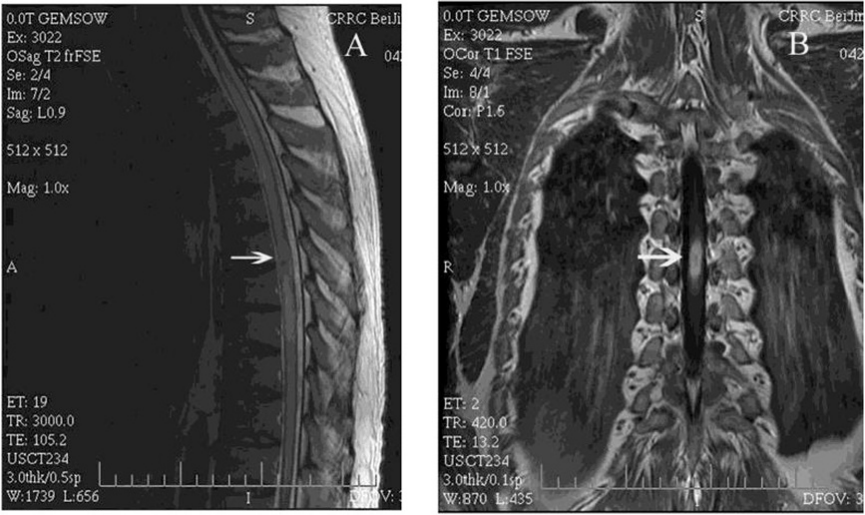
**The MRI images of the thoracic spine at the time of neurologic deterioration. A)** Sagittal T_2_-weighted MRI image shows an increase in the size of the lesion. **B)** Coronal T_1_-weighted MRI image shows an enlarged ring-like enhancing intramedullary lesion (40 × 10 × 7 mm) with gadolinium.

**Figure 3:**
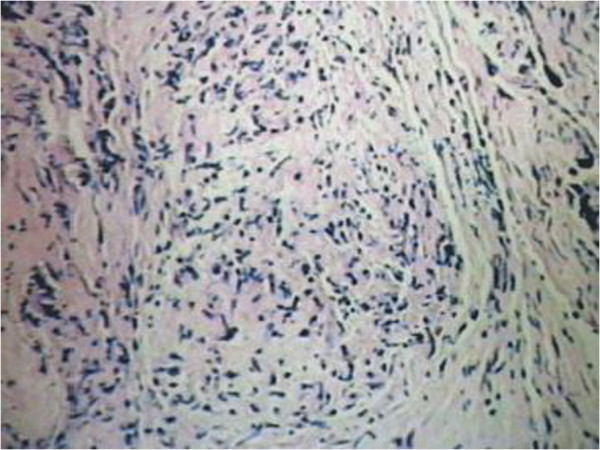
**Histological examination showing epithelioid cells, lymphocytes, and Langerhans giant cells indicative of tuberculoma (hematoxylin-eosin, original magnification × 10).**

After admission the patient was re-examined. MRI of the thoracic spine showed a hyperintense lesion expanding the cord from T_6_ through T_7_ on T_2_-weighted images, and on T_1_-weighted images there was an isointense lesion expanding the cord from T_6_ through T_7_ with ring enhancement after gadolinium diethylenetriaminepentaacetic acid (GDTA) administration (Figure 1A–C). Results of cerebrospinal fluid (CSF) analysis were normal and the CSF culture did not grow any bacteria. A tuberculin skin test was negative. Chest radiography revealed no abnormality. Therefore, spinal cord infarction (SCI), intramedullary inflamed granuloma, or intramedullary neoplasm was considered to be the cause of paraplegia following epidural anesthesia. The patient was treated with dexamethasone, antiviral medications, and rehabilitation training. These therapies apparently relieved his neurologic symptoms. However, 20 days after administration his sensorimotor functions suddenly deteriorated. Numbness and paresthesia sensation extended up to the T_5_ levels. Muscle power of the lower limbs decreased to grade 0/5 over the right lower limb and 2/5 over the left lower limb. MRI studies at this time showed an enlargement of the intramedullary lesion (Figure 2A, B). Because of the patient’s neurologic deterioration and the uncertain diagnosis, complete surgical excision of the intramedullary mass was performed. Histopathologic examination revealed a tuberculous granulomatous lesion (Figure 3). Antituberculous treatment was initiated with rifampicin, isoniazid, and pyrazinamide after surgical intervention and was continued for 6 months. At the last follow-up visit, 7 months after laminectomy, his motor and sensory functions had gradually improved over time. He was able to walk with assistance and had regained bladder control.

### Discussion

Paraplegia is a rare but tragic complication following epidural anesthesia. When postoperative paraplegia occurs, the anesthetic technique, surgical procedure, and coexisting disease should all be considered. In this case, the postoperative paraplegia was related to intramedullary spinal tuberculoma. However, we were unable to ascertain the exact cause of paraplegia before histopathologic examination. From clinical symptoms, laboratory tests, and imaging studies, the possible causes of postoperative paraplegia in this patient included acute transverse myelitis, SCI, and intramedullary inflamed granuloma or neoplasm.

Acute transverse myelitis (ATM) represents a major subset of acute noncompressive myelopathies characterized by acute or subacute dysfunctions of motor, sensory, and autonomic nerves [2], with an incidence of 1–4 per 1 million persons per year. Individual sporadic cases of transverse myelitis have been reported following spinal, epidural, or general anesthesia, but the causative agent has not been identified [3]. Because the clinical symptoms and abnormal gadolinium enhancement of the spinal cord at MRI were noted in our patient, the diagnosis of ATM could not be excluded despite normal CSF analysis.

SCI is a rare disorder characterized by flaccid paraplegia with absent deep tendon reflexes, but with preserved proprioception and vibration sense. It has been reported that the causes of SCI following epidural anesthesia include compression of the spinal arteries by the volume of epidural solution, systemic hypotension, pre-existing vascular disease, the local vasoconstrictor effect of epinephrine, and possible acute thrombosis of the anterior spinal or radicular arteries [4]. In this case, introducing an excessive amount of anesthetics into the epidural space and using epinephrine in the local anesthetic solution were possible contributory risk factors. However, the absence of hypotension during the operation, the loss of positional sense in the patient’s left leg, and the characteristics of signal abnormity on MRI examination do not completely support the diagnosis of SCI.

Intramedullary spinal tuberculoma is a rare form of central nervous system tuberculosis, which occurs usually in young people and in the thoracic spinal cord. Although it frequently presents signs of subacute spinal cord compression, variable clinical manifestations including Brown-Sequard syndrome and episodes of paraplegia have also been reported [5]. The MRI of intramedullary tuberculomas shows gadolinium ring enhancement, with or without central hyperintensity on T2-weighted images, and gadolinium to isointense rings on T1-weighted images [6]. In our patient there was an isointense area with cord expansion on T1-weighted images and a hyperintense area on T2-weighted images, with ring enhancement after contrast administration. Unfortunately, the diagnosis of intramedullary spinal tuberculoma was delayed despite the typical imaging features, for several possible reasons. First, the patient had no history of tuberculosis. It has been suggested that intramedullary tuberculomas are almost always associated with active pulmonary tuberculosis [7]. Second, the tuberculin skin test, chest radiography, and CSF examinations were all normal. Lastly, postoperative paraplegia accidently occurred following epidural anesthesia, and the puncture site was adjacent to the lesion location of intramedullary spinal tuberculoma. Therefore, it was necessary to identify anesthesia-related complications.

The optimal treatment of intramedullary tuberculoma remains debatable. Both surgical and medical treatments have been reported to achieve reasonable efficacy in different studies [7–9]. Medical treatment includes antituberculous chemotherapy and steroid therapy. The addition of corticosteroids with single or multiple tuberculomas resulted in an improved clinical outcome, presumably by reducing edema. For this reason, the neurologic function of our patient was improved temporarily after dexamethasone treatment. Surgery is generally indicated when (1) there is no response to chemotherapy, (2) the diagnosis is in doubt, and (3) there are large lesions with rapid deterioration in neurologic function [7]. Because of the uncertain diagnosis and deterioration of neurologic status, surgical intervention was performed in our patient. Despite subsequent antituberculous therapy, the patient retained some disability.

## Conclusion

The cause of postoperative paraplegia in this patient was intramedullary spinal tuberculoma. When a neurologic injury is diagnosed postoperatively, differential diagnostic approaches must include identification of any coexisting disease. Although MRI is the most sensitive diagnostic test for establishing the actual diagnosis, it is still difficult to differentiate SCI, myelitis, intramedullary spinal tuberculoma, and neoplasms from imaging features alone.

## Consent

Written informed consent was obtained from the patient for publication of this case report and any accompanying images. A copy of the written consent is available for review by the Editor of this journal.

## Abbreviations

CT: Computed tomography
MRI: Magnetic resonance imaging
GDTA: Gadolinium diethylenetriaminepentaacetic acid
CSF: Cerebrospinal fluid
ATM: Acute transverse myelitis
SCI: Spinal cord infarction.
